# Multiple solitary fibrous tumors of the pleura with multicentric and unilateral involvement: a case report

**DOI:** 10.1186/s40792-023-01717-5

**Published:** 2023-07-26

**Authors:** Takuya Watanabe, Eriko Suzuki, Naoko Yoshii, Takuya Kohama, Kensuke Iguchi, Suiha Takeuchi, Minori Nakamura, Takumi Endo, Masayuki Tanahashi

**Affiliations:** Division of Thoracic Surgery, Respiratory Disease Center, Seirei Mikatahara General Hospital, 3453, Mikatahara-cho, Kita-ku Hamamatsu, Shizuoka, Japan

**Keywords:** Solitary fibrous tumor, Pleura, Thoracic, Multiple, Multicentric

## Abstract

**Background:**

Solitary fibrous tumor of the pleura (SFTP) is a mesenchymal tumor. Patients with SFTP generally have only one lesion. We herein report an extremely rare case of multiple SFTPs that were multicentric and unilateral.

**Case presentation:**

The patient was a 21-year-old asymptomatic young man who was referred to our hospital due to abnormal shadows on a chest X-ray. Computed tomography showed 6 tumors of heterogeneous sizes in the left thoracic cavity. The tumors were suspected to be multiple benign or low-grade malignant thoracic tumors, and tumor resection was performed. The tumors had almost the same appearance, with uniform fibroblastic spindle cell proliferation, and arose from the pleura in microscopy. Immunohistochemical staining revealed that the tumor cells were positive for CD34, CD99, Bcl-2, and STAT6. Based on these findings, the tumors were diagnosed as multiple SFTPs with multicentricity. At 1 year and 6 months after the first surgery, 2 new lesions were found above the diaphragm, and these were resected. These tumors were arose from the pleura with a fibrous capsule structure. Their pathological findings were identical to the initial tumor without evidence of malignant transformation.

**Conclusion:**

We experienced an extremely rare case of multiple SFTPs with multicentric and unilateral lesions.

## Background

Solitary fibrous tumors of the pleura (SFTPs) are relatively rare, accounting for < 5% of all pleural tumors [1]. Although there are differences in tumor size and clinicopathological findings, patients with SFTP generally have only one lesion [2, 3]. Recent reviews of SFTPs have not described a case of multiple SFTPs [4, 5]. In addition, SFTPs are commonly present during the fifth and sixth decades of life [6, 7]. In this background, we report an extremely rare case of multiple SFTPs with multicentric and unilateral lesions in a young patient.

## Case presentation

**Fig. 1:**
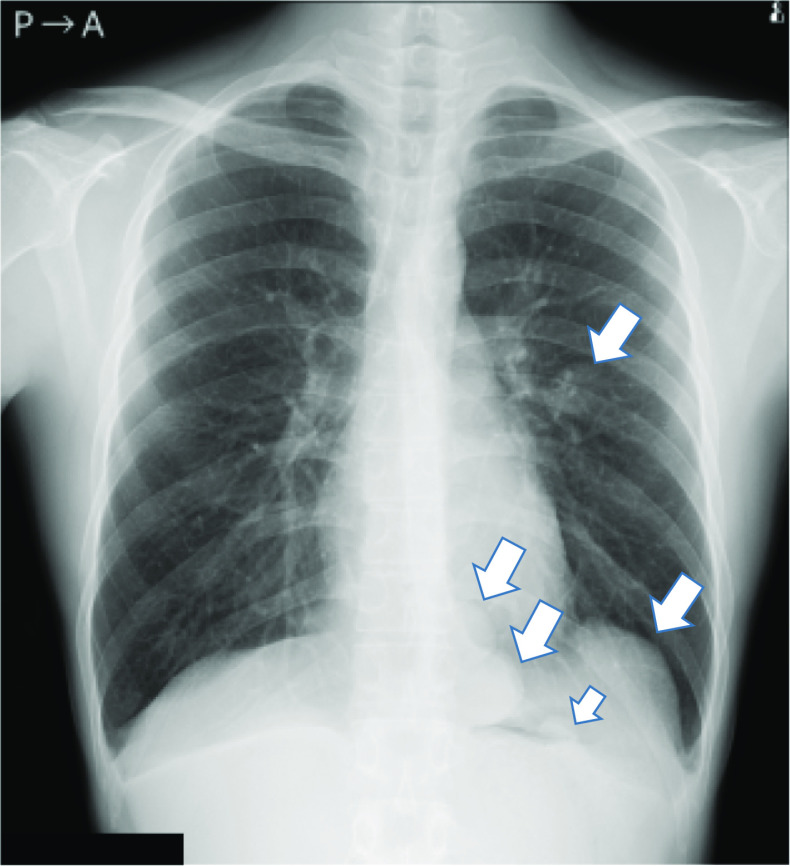
A chest radiograph showed several lesions in the left lung field (arrows)

**Fig. 2:**
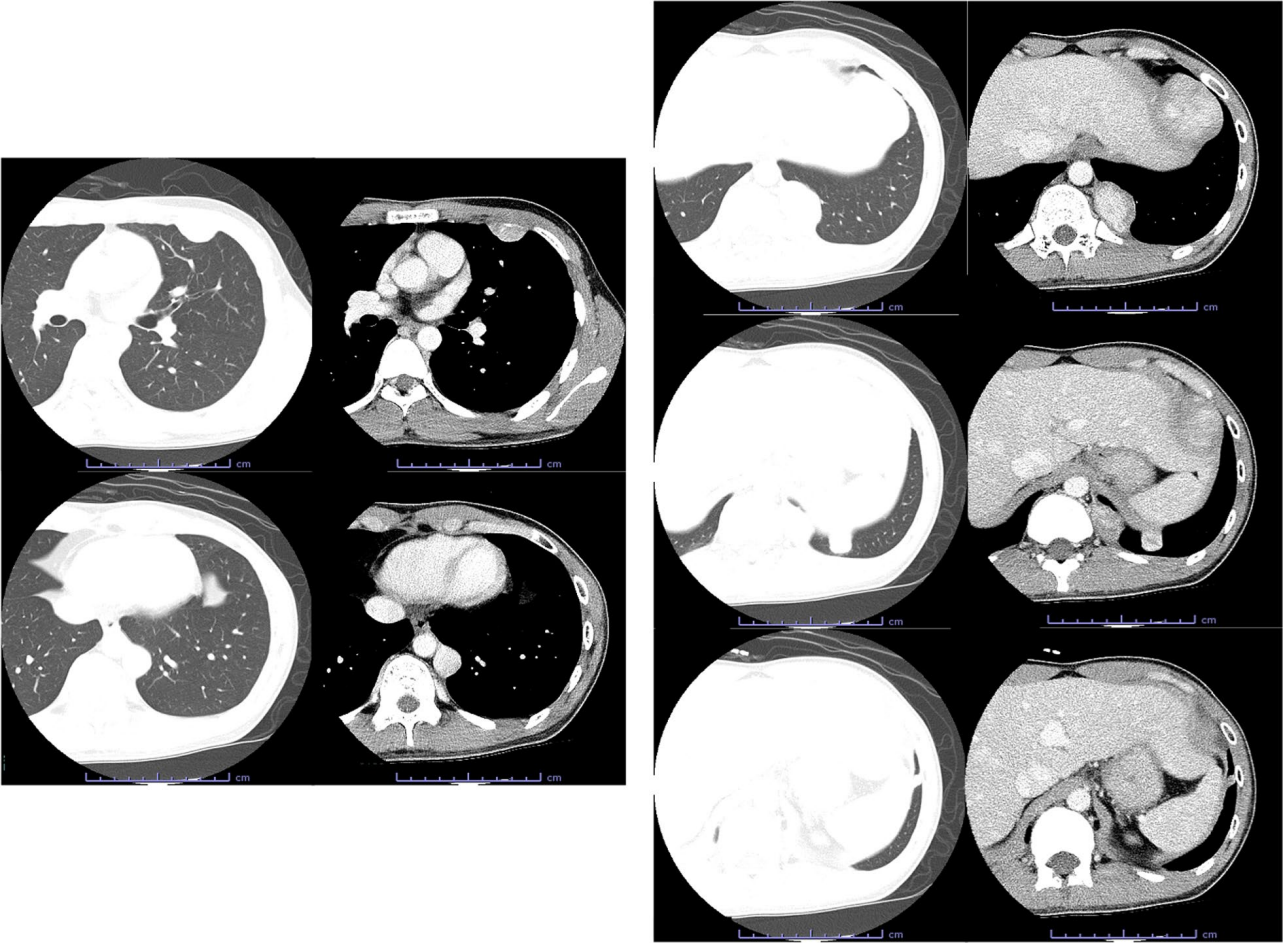
Chest CT showed 6 tumors in the left thoracic cavity. These tumors were well defined, noninvasive, lobular, noncalcified, adjacent to the chest wall, and unevenly enhanced on contrast-enhanced CT

**Fig. 3:**
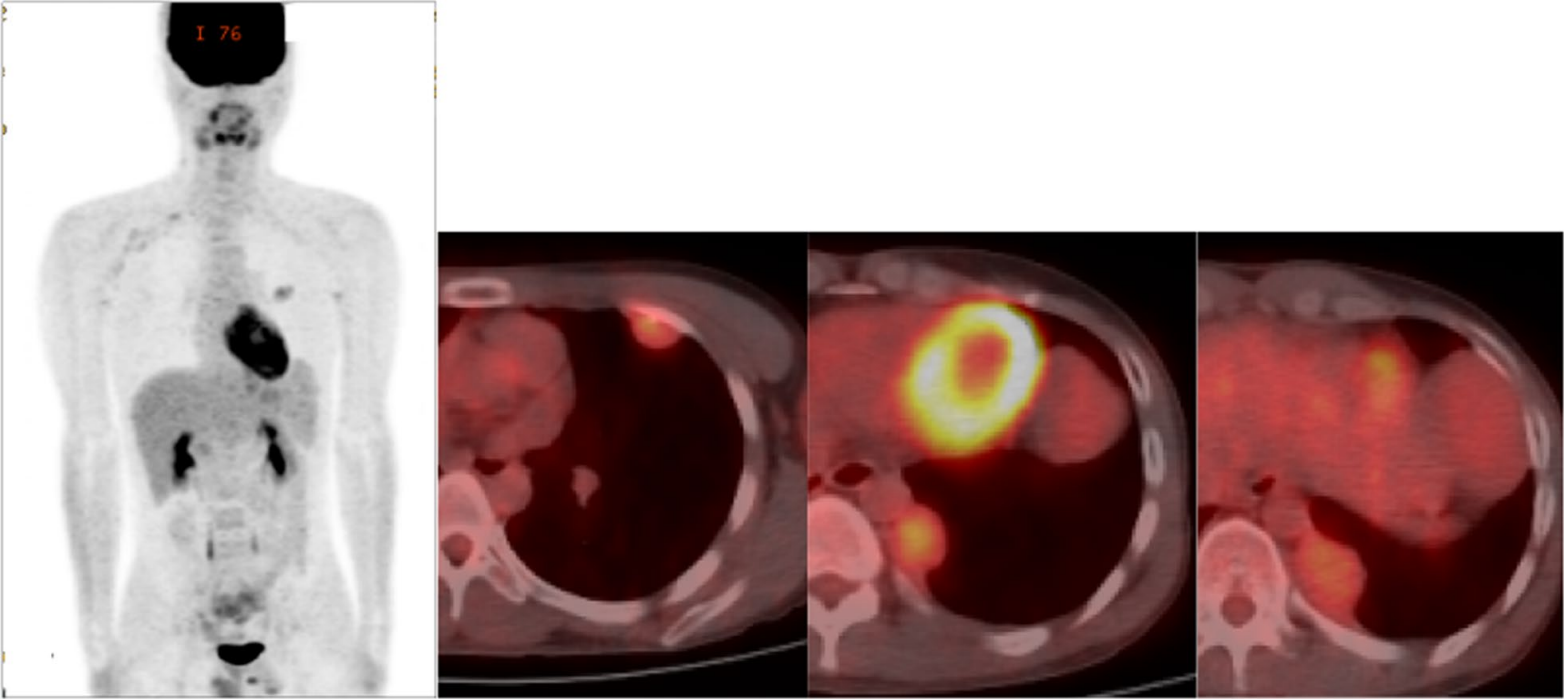
FDG-PET showed an SUVmax of 0.5 to 3.2 within each tumor

The patient was a 21-year-old asymptomatic young man who was referred to our hospital due to abnormal shadows on chest X-ray (Fig. 1). Chest computed tomography (CT) showed 6 tumors of 1 cm to 5.5 cm in size located in the left thoracic cavity (Fig. 2). These tumors were well defined, noninvasive, lobular, noncalcified, adjacent to the chest wall, and unevenly enhanced on contrast-enhanced CT. No tumors were found in the right thoracic cavity. ^18^Fluorodeoxyglucose-positron emission tomography (FDG-PET) showed maximum standardized uptake values (SUVmax) of 0.5 to 3.2 within each tumor (Fig. 3). The tumors were suspected to be multiple benign or low-grade malignant thoracic tumors, and tumor resection was performed for the diagnostic and therapeutic purposes.

**Fig. 4:**
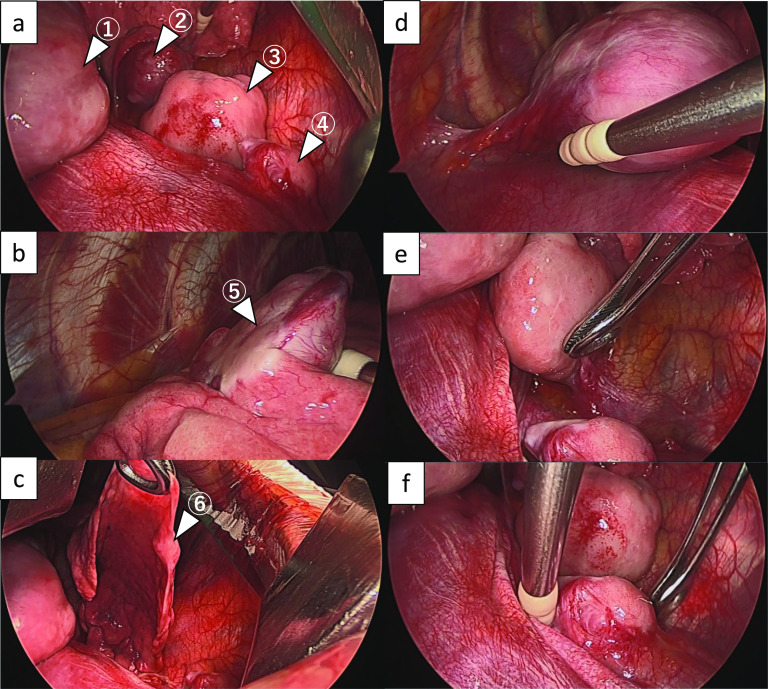
The intraoperative findings. Four tumors arose from the parietal pleura (**a**), and 2 arose from the visceral pleura (**b**, **c**). Two tumors were connected to the pleura with a thin stalk (**d**, **e**), and one had a wide base (**f**)

Four tumors arose from the parietal pleura (Fig. 4a), and 2 arose from the visceral pleura (Fig. 4b, c). Two tumors were connected to the pleura with a thin stalk (Fig. 4d, e), and one had a wide base (Fig. 4f). One tumor needed combined resection of the diaphragm, and two needed combined partial resection of the lung. The diaphragm, lungs, and chest wall were closely observed during surgery, and there were no residual lesions.

**Fig. 5:**
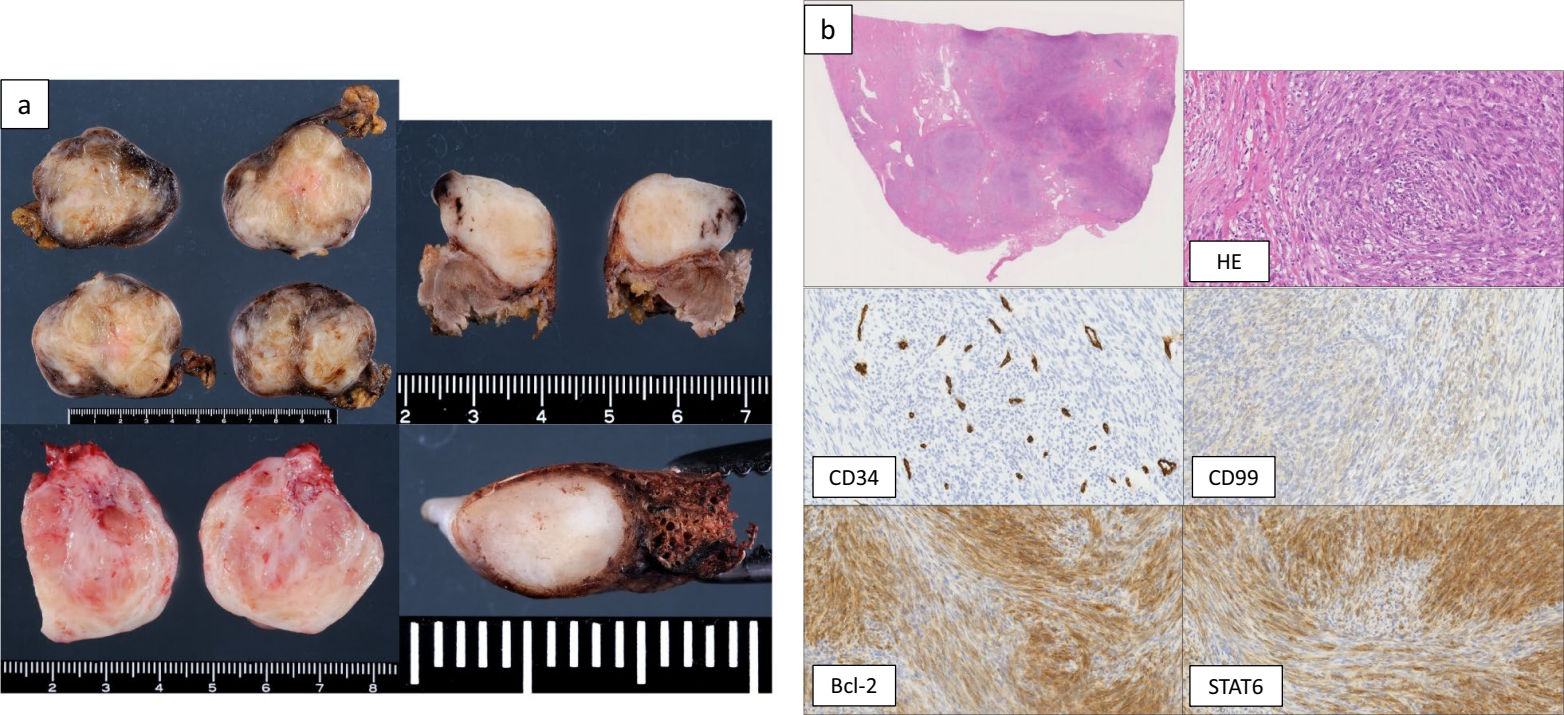
The histopathological findings of the resected tumors. The tumors were of heterogeneous size, ranging from 1 cm to 5.7 cm. A macroscopic examination revealed that they had almost the same appearance (whitish, elastic soft, round, and lobular (**a**). Microscopy revealed uniform fibroblastic spindle cell proliferations arising from the pleura, and immunohistochemical staining revealed that the tumor cells were positive for CD34, CD99, Bcl-2, and STAT6 (**b**)

The tumors ranged from 1 cm to 5.7 cm in size, and had almost the same macroscopic appearance (whitish, elastic soft, round shape and lobular) (Fig. 5a). Microscopy revealed uniform fibroblastic spindle cell proliferation arising from the pleura (Fig. 5b). Immunohistochemical staining revealed that the tumor cells were positive for CD34, CD99, Bcl-2, and STAT6 (Fig. 5b). Based on these findings, all of the tumors were diagnosed as SFTP. The tumors had a membrane of fibrofatty stromal tissue, and no invasion was found in the combined resected diaphragm and lungs. The mitosis counts were 0–1/10 in high-power fields, and no necrosis was found. Therefore, the tumor was judged to have a low risk of recurrence. The postoperative course was good without complications.

**Fig. 6:**
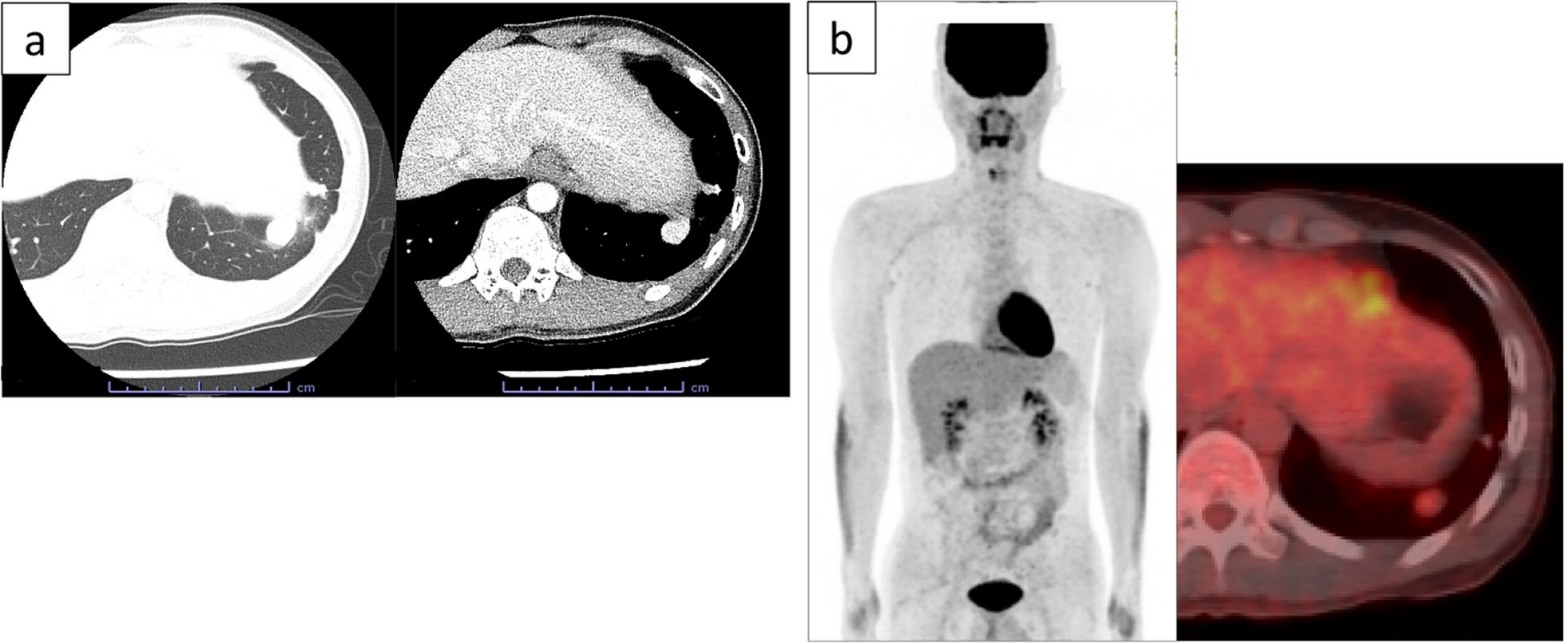
Two new lesions above the diaphragm were found on chest CT at 1 year and 6 months after the first surgery (**a**). FDG-PET showed that the SUVmax intake of the tumors was 2.32, and metastasis to other organs was not found (**b**)

**Fig. 7:**
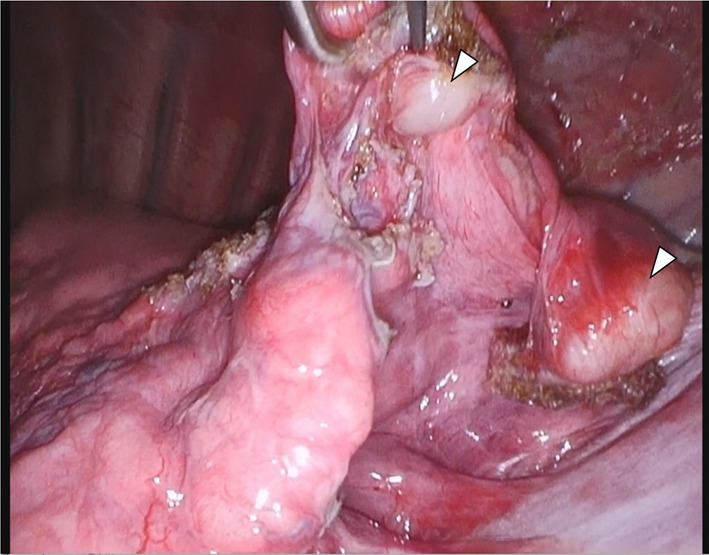
Intraoperative findings of the second surgery. One tumor was found in the left lower lobe. The other was on the diaphragm (arrowheads)

**Fig. 8:**
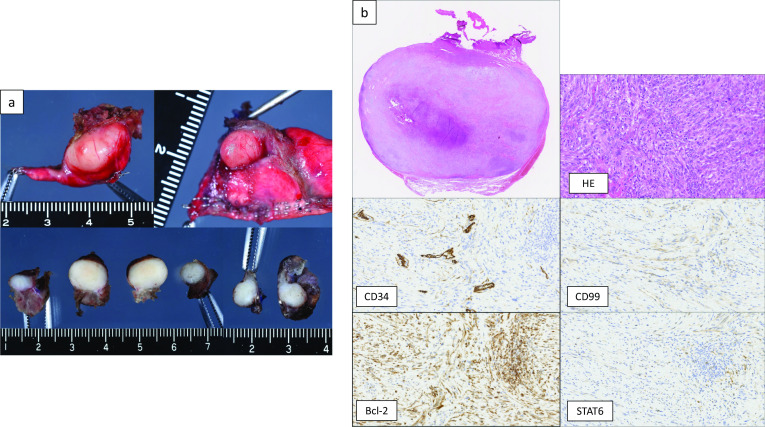
The histopathological findings of the resected tumors by the second surgery. The same pathological findings were revealed for tumors resected by the first surgery, including immunohistochemical positivity for CD34, CD99, Bcl-2, and STAT6 (**a**, **b**)

At 1 year and 6 months after the first surgery, chest CT revealed 2 new lesions above the diaphragm (Fig. 6a). Retrospective evaluation revealed that these were not present on the chest CT scans obtained at the time of the first procedure. FDG-PET showed that the SUVmax intake of the tumors was 2.32 (Fig. 6b), and metastasis to the other organs was not found. Tumor resection was performed. One tumor was found in the left lower lobe, and the other was found on the diaphragm (Fig. 7). The pathological findings were consistent with those observed at the time of the first surgery (Fig. 8a, b). The new tumors were diagnosed as SFTP. Since these tumors arose from the pleura, had a fibrous capsule structure, and showed no evidence of malignant transformation, they were diagnosed as de novo tumors rather than disseminated or recurrent tumors. The postoperative course was also good, without complications or recurrence in the 2 months after the second surgery.

## Discussion

SFTPs are relatively rare tumors, with an incidence of 1 new patient per million people per year [4], and account for < 5% of all pleural tumors [1]. The incidence of SFTPs does not differ between the sexes and SFTPs occur in all age groups; however, they commonly present during the fifth and sixth decades of life [6, 7].

Half of the patients (43% to 67%) are asymptomatic at the time of the diagnosis [8–10]. Larger and malignant SFTPs are more likely to be associated with symptoms such as cough, chest pain, or dyspnea [11–14]. In our case, the patient had no symptoms, despite having many tumors, which may have been because the tumors were < 5.0 cm. Immunostaining is useful in the diagnosis of SFTP, and CD34, CD99, and Bcl-2 are commonly used as positive markers. In addition, NAB2-STAT6 gene fusion has recently emerged as a sensitive and specific molecular marker and its IHC surrogate marker signal transducer and activator of transcription 6 (STAT6) has also shown significant sensitivity and specificity for the diagnosis of SFTP [6]. In our case, it was easy to make an individual diagnosis because these markers were all positive.

The chest CT findings in this case matched the features of SFTP, including a homogeneous well-defined, noninvasive, lobular, soft-tissue mass adjacent to the chest wall or within a fissure [8, 9]. However, multiple tumors were present, and if they were SFTPs, they would be considered to have malignant potential. On the other hand, on FDG-PET, malignant SFTPs are reported to have a median SUVmax of 3.6 (range 2.5–4.9) [15, 16], and this patient’s SUVmax was relatively lower in comparison to other reports. After surgery, these were diagnosed as multiple SFTPs with low risk of recurrence based on a pathological examination because there were few (or no) mitoses, and no necrosis. In addition, each tumor arose from the pleura, and these findings proved that they were not disseminated from one tumor and were multicentric. It was considered that the new lesions found at 1 year and 6 months after the first surgery might have recurred from one of the resected tumors. However, they were diagnosed as newly arising tumors rather than recurrence, dissemination or, malignant change based on the pathological findings. On the other hand, at the time of the second surgery, the tumors had been growing at 1 year and 6 months. It is unclear why they grew in a relatively short period of time, since their pathology showed only one to five fission images per field of view, which is not very many. It is possible that the invasiveness of the initial surgery had some effect on the tumor growth.

Furthermore, these tumors were found in only the left thoracic cavity. This has not been described in multicenter cohorts, meta-analyses, or systematic reviews [3–5]. Although there is one report of a patient with multiple SFTs, it was malignant, and tumors were not limited to the thoracic region, they were found throughout the body [16]. In contrast to many of the reports on SFTP, our case was considered extremely rare. There were no reports similar to ours as far as we could find in PubMed and Scopus. To the best of our knowledge, this is the first case report of SFTPs with unilateral and multicentric. We also reported a rare case of atypical SFTP with cystic degeneration [17] and describe another rare case of SFTP with multiple, multicentric, and unilateral lesions in the present report. Based on these experiences, it seems that there is still uncharted territory in SFTP.

Finally, this patient was young, which is a risk factor for metastasis and mortality [18], and he had new tumors in the short term after the first surgery. There may be invisible lesions in the thoracic cavity yet. Although all of the resected tumors were low-grade malignancies, additional lesions that may have malignant changes may be found in the future; thus, we are continuing to closely observe the patient.

## Conclusion

We reported an extremely rare case of multiple SFTPs that were multicentric and unilateral.

## Data Availability

Not applicable.

## Abbreviations

SFTP: Solitary fibrous tumor of the pleura
CT: Computed tomography
FDG-PET: 18Fluorodeoxyglucose-positron emission tomography
SUVmax: Maximum standardized uptake values
